# Human milk oligosaccharide 2’-fucosyllactose links feedings at 1 month to cognitive development at 24 months in infants of normal and overweight mothers

**DOI:** 10.1371/journal.pone.0228323

**Published:** 2020-02-12

**Authors:** Paige K. Berger, Jasmine F. Plows, Roshonda B. Jones, Tanya L. Alderete, Chloe Yonemitsu, Marie Poulsen, Ji Hoon Ryoo, Bradley S. Peterson, Lars Bode, Michael I. Goran

**Affiliations:** 1 Department of Pediatrics, The Saban Research Institute, Children’s Hospital Los Angeles, Los Angeles, California, United States of America; 2 Department of Integrative Physiology, University of Colorado Boulder, Boulder, Colorado, United States of America; 3 Department of Pediatrics and Mother-Milk-Infant Center of Research Excellence, University of California, San Diego, La Jolla, California, United States of America; 4 University Center for Excellence in Developmental Disabilities, Children’s Hospital Los Angeles, Los Angeles, California, United States of America; Texas A&M University College Station, UNITED STATES

## Abstract

**Background:**

Infant cognitive development is influenced by maternal factors that range from obesity to early feeding and breast milk composition. Animal studies suggest a role for human milk oligosaccharide (HMO), 2’-fucosyllactose (2’FL), on learning and memory, yet no human studies have examined its impact on infant cognitive development relative to other HMOs and maternal factors.

**Objective:**

To determine the impact of 2’FL from breast milk feeding on infant cognitive development at 24 months of age relative to maternal obesity and breast milk feeding frequency.

**Methods and materials:**

Hispanic mother-infant pairs (N = 50) were recruited across the spectrum of pre-pregnancy BMI. Breast milk was collected at 1 and 6 months, and feedings/day were reported. Nineteen HMOs were analyzed using high-performance liquid chromatography, with initial interest in 2’FL. Infant cognitive development score was assessed with the Bayley-III Scale at 24 months. Linear regressions were used for prediction, and bootstrapping to determine mediation by 2’FL.

**Results:**

Maternal pre-pregnancy BMI was not related to feedings/day or HMOs, but predicted poorer infant cognitive development (β = -0.31, P = 0.03). Feedings/day (β = 0.34) and 2’FL (β = 0.59) at 1 month predicted better infant cognitive development (both P≤ 0.01). The association of feedings/day with infant cognitive development was no longer significant after further adjustment for 2’FL (estimated mediation effect = 0.13, P = 0.04). There were no associations of feedings/day and 2’FL at 6 months with infant cognitive development.

**Conclusions:**

Our findings suggest that maternal factors influence infant cognitive development through multiple means. Though maternal obesity may be a separate negative influence, greater frequency of breast milk feeding at 1 month contributed to infant cognitive development through greater exposure to 2’FL relative to other HMOs. The influence of 2’FL was not significant at 6 months, indicating that early exposure to 2’FL may be a critical temporal window for positively influencing infant cognitive development.

## Introduction

Infancy is a critical period of brain organization and plasticity that supports cognitive development. During the first 6 months of postnatal life in particular, infants undergo rapid brain growth that places an unusually high demand on the available pool of nutrients and biochemical building blocks for cognitive development processes [1]. This is a window of opportunity to support external influences that can optimize brain maturation and cognitive development, as early dietary exposures have the potential to mitigate cognitive deficits and maximize learning and memory for long-term brain function and health [2–4].

It follows that this critical period of brain plasticity coincides with the window of recommended exclusive breast milk feeding. The benefits of breast milk feeding have been well documented: infants exposed to a longer duration of breast milk feeding had higher intelligence test scores in childhood [5], an outcome that is stable into adulthood [6]. However, the magnitude and extent to which breast milk feeding is beneficial varies across studies [7]. This may be attributed to several factors. First, maternal characteristics contribute to breast milk characteristics: mothers who are obese may have more difficulty with breast milk expression to meet the demands of the infant [8]. Moreover, maternal obesity alters breast milk composition: this includes neuroactive components, e.g., leptin and adiponectin [9]. Exposure to these compounds has been shown to influence infant cognitive performance [10, 11]. It stands to reason that maternal obesity and breast milk feeding frequency may impact degree of exposure to neuroactive components at critical stages of brain maturation, and this may in turn influence infant cognitive development.

Despite this knowledge, there is a lack of current data on specific breast milk components that optimize infant cognitive development and learning potential. One component that may be relevant is a group of non-digestible carbohydrates known as human milk oligosaccharides (HMOs), the third most predominant component of breast milk [12]. Of the more than 150 HMOs that have been identified so far, 2’-fucosyllactose (2’FL) may be the most promising candidate for positively influencing infant cognitive abilities. Animal studies revealed that exposure to 2’FL enhanced cognitive outcomes of learning, memory, and attention in rodents [13, 14]. This may be attributed to several mechanisms. For example, HMOs are a source of prebiotics to nourish the gut microbiome, which ferments these molecules into metabolites that regulate brain signaling, and diminishes gut dysbiosis (imbalance) and inflammation that can cause brain injury [15, 16]: in vitro, 2’FL increased abundance of gut microbes *Bacteroides* and *Lactobacillus* [17], and a greater abundance of the same gut microbes was related to better cognitive abilities in infants [18].

Though maternal and breast milk characteristics may be mutually beneficial (or harmful), the extent to which each exposure may impact infant cognitive abilities is unclear and a topic of much debate. Therefore, the aim of this study was to determine the influence of 2’FL from breast milk feeding on infant cognitive development by 24 months of age relative to maternal obesity and breast milk feeding frequency.

## Materials and methods

### Subjects

Participants in this study were 50 mother-infant pairs recruited from maternity clinics in Los Angeles County. As described in Berger et al., mothers were included based on the following criteria: 1) self-identified Hispanic ethnicity; 2) ≥18 years old at delivery; 3) gave birth to a healthy, term, singleton newborn; 4) enrolled within 1 month postpartum; 5) intended to breastfeed for 6 months postpartum; and 6) able to read English or Spanish at a 5^th^ grade level to understand procedures [19]. Mothers reported pre-pregnancy weight and height so as to recruit uniformly across the spectrum of pre-pregnancy BMI status [20]. Mothers were excluded based on the following criteria: 1) reported medications or a medical condition that could affect physical or mental health, nutrition, or metabolism; 2) used tobacco (>1 cigarette/week) or recreational drugs; and 3) had a clinical diagnosis of fetal abnormalities [19]. The Institutional Review Board of Children’s Hospital Los Angeles and the University of Southern California approved all procedures. Participants provided written informed consent prior to data collection [19].

### Study design

Mother-infant pairs completed three visits for the purposes of this study. As described in Berger et al. at 1 month, historical health-related information was collected, and included maternal age, infant sex, and infant birth weight [19]. At 1 and 6 months, mothers completed questionnaires on breast milk feeding practices: mothers were asked to report mean breast milk feedings/day (i.e., breast milk feeding frequency) for the past 7 days. At 1, 6, and 24 months, infant weight was measured following standard procedures [19]. Mothers were weighed with and without holding the infant on an electronic scale, and the difference in the mother’s weight with and without holding the infant was calculated and recorded [19].

### Breast milk collection and HMO analysis

At 1 and 6 months, breast milk was collected and analyzed following standard procedures [21–23]. Mothers were instructed to refrain from eating for 1 hour and feeding and/or pumping breast milk for 1.5 hours beforehand. Mothers were encouraged to pump the entire contents of a single breast milk expression. Aliquots were stored at -80°C until HMO analysis at the University of California San Diego. Raffinose was added to each sample as an internal standard for absolute quantification. HMOs were isolated with high-throughput solid-phase extraction, fluorescently labeled, and measured using high-performance liquid chromatography [23]. Nineteen HMOs were quantified based on standard retention times and mass spectrometric analysis. These individual HMOs account for >90% of total HMO composition, and include the following: 2’FL, 3-fucosyllactose (3FL), 3’-sialyllactose (3’SL), 6’-sialyllactose (6’SL), difucosyllactose (DFLac), lacto-N-tetraose (LNT), lacto-N-neotetraose (LNnT), lacto-N-fucopentaose (LNFP) I, LNFPII, LNFPIII, sialyl-LNT (LST) b, LSTc, difucosyl-LNT (DFLNT), disialyl-LNT (DSLNT), lacto-N-hexaose (LNH), fucosyl-LNH (FLNH), difucosyl-LNH (DFLNH), fucosyl-disialyl-LNH (FDSLNH), and disialyl-LNH (DSLNH). Secretor status was defined by the presence or near absence of HMOs 2’FL or LNFP I, and used as a control variable [23].

### Infant cognitive development assessment

The Bayley Scales of Infant Development (Third Edition, Bayley-III) was administered by trained research personnel to assess the developmental functioning of cognitive, language, and motor skills at 24 months of age [24]. The Bayley-III cognitive scale measures sensorimotor integration, concept formation, attention, habituation, and memory. Based on the premise of this study [13, 25], analyses were limited to the cognitive scale from this assessment. Scaled (age-standardized) scores, rather than raw scores, for cognitive development abilities were used as the dependent (or outcome) variable. The scaled score is a transformation of the raw score to the average performance of a normative sample at a given age. It corresponds to a set position on the normal distribution curve [24]. The internal consistency of the Bayley-III cognitive scale is 0.91 [26].

### Statistical analysis

Descriptive statistics are presented as mean ± standard deviation (SD) for continuous variables and as percentages for categorical variables [19]. Normal distribution and homogeneity of variances were confirmed using Shapiro–Wilks *W* and Levene’s tests, respectively. Differences between maternal pre-pregnancy BMI status groups were tested using analysis of variance with polynomial contrast for continuous variables and by Mantel–Haenszel linear-by-linear association chi-square tests for categorical variables [19]. In addition, changes in individual HMOs from 1 month to 6 months that were associated with the outcome variable were determined with paired samples t-tests.

As described in Berger et al., we conducted a mediation analysis [19]. Mediation analysis posits that the relationship between exposure and outcome is mediated by a mechanism factor: the mediator [19]. To be considered a mediator, the following criteria must be met: 1) the exposure variable is significantly related to the outcome variable (Path C); 2) the exposure variable is significantly related to the mediator variable (Path A); 3) the mediator variable is significantly related to the outcome variable, after adjustment for the exposure variable (Path B); and 4) after adjustment for the potential mediator, a previously significant association between the exposure variable and outcome variable is no longer significant (Path C`) [19, 27]. We performed linear regression analyses to examine the following relationships: 1) between breast milk feeding frequency (exposure) and infant cognitive development scores (outcome); and 2) between breast milk feeding frequency and the potential mediator (HMO 2’FL). We then examined the extent to which the association between breast milk feeding frequency and infant cognitive development scores was explained (i.e., potentially mediated) by the potential mediator considered [19]. This was done by quantifying the attenuations in the magnitude of the linear regression coefficient reflecting the association between breast milk feeding frequency and infant cognitive development scores after adjustment for the HMO 2’FL measurement. Before analyses, the distributions of breast milk feeding frequency, HMO 2’FL, and infant cognitive development scores were assessed and found to be normal. We also confirmed that the residuals of the outcome, exposure, and mediator variables were independent of each other and that there was no misspecification of causal order or misspecification of causal direction: infant cognitive development scores (outcome) could not affect breast milk feeding frequency (exposure) or HMO 2’FL (mediator) [19]. The mediation effect was tested for significance using the bootstrap method. The same analyses were conducted replacing breast milk feeding frequency with maternal pre-pregnancy BMI as the exposure variable. All models were adjusted for the following covariates: maternal secretor status, age at delivery, education level, infant sex, age, and birth weight. All analyses were conducted with SPSS software (version 24; IBM SPSS Statistics, Chicago, IL). Statistical significance was set at a two-tailed *P* value <0.05.

## Results

The sample was composed of 50 Hispanic mother-infant pairs. In total, mothers were 30.6 ± 7.1 years old at delivery, had a pre-pregnancy BMI of 27.5 ± 5.8 kg/m2, were 86.0% secretors, and were 76% high school graduates. The percentages of mothers who were normal weight, overweight, and obese were 34.0%, 36.0%, and 30.0%, respectively. Characteristics of the mother-infant dyads grouped by maternal pre-pregnancy BMI status are described in Table 1. Distribution of infant sex, age, birth weight, and weight at 1, 6, and 24 months did not differ between groups. In addition, there were changes in individual HMOs over lactation. Though 2’FL and LSTb were similar from 1 month to 6 months (both P = 0.50), DSLNT (435 ± 181 vs. 365 ± 207 ug/mL), LNH (108 ± 56 vs. 70.0 ± 47 ug/mL), and FLNH (155 ± 93 vs. 57.8 ± 71 ug/mL) decreased from 1 month to 6 months (P≤ 0.01).

**Table 1:** Characteristics of the Hispanic mother-infant pairs.

V	Maternal pre-pregnancy BMI status^a^				*P*^b^
	Total	Normal weight	Overweight	Obese	
*n*	50	17	18	15	
Mothers					
Age at delivery (years)	30.6 ± 7.1	31.3 ± 7.1	28.7 ± 7.9	32.0 ± 7.1	0.38
BMI, pre-pregnancy (kg/m^2^)	27.5 ± 5.8	21.5 ± 1.4	27.4 ± 1.2	34.5 ± 4.3	<0.01
Caesarean section delivery (%)^c^	25.1	25.4	20.3	29.3	0.46
Secretor (%)^c^	86.0	85.4	83.6	88.9	0.73
Education level (%)^c^					0.38
<8^th^ grade	8.00	0.00	16.7	6.70	
Completed 8^th^ grade	2.00	0.00	0.00	6.70	
Some high school	14.0	17.6	11.1	13.3	
Completed high school	28.0	29.4	22.2	33.3	
Some college	26.0	11.8	38.9	26.7	
Completed college	12.0	23.5	5.60	6.70	
Completed graduate school	10.0	17.6	5.60	6.70	
Infants					
Female (%)^c^	53.8	57.9	50.8	52.5	0.68
Age (days)	728 ± 54	719 ± 93	735 ± 11	732 ± 9.5	0.65
Birth weight (kg)	3.31 ± 0.3	3.29 ± 0.3	3.27 ± 0.2	3.37 ± 0.4	0.61
Breast milk feedings/day, 1 month (number)	7.16 ± 2.0	7.58 ± 1.2	7.00 ± 2.1	6.87 ± 2.6	0.56
Breast milk feedings/day, 6 months (number)	4.02 ± 3.2	4.69 ± 3.2	3.11 ± 3.4	4.40 ± 2.9	0.32
Weight, 1 month (kg)	4.58 ± 0.4	4.55 ± 0.5	4.68 ± 0.3	4.49 ± 0.3	0.42
Weight, 6 months (kg)	7.87 ± 0.7	7.86 ± 0.8	7.88 ± 0.6	7.85 ± 0.6	0.99
Weight, 24 months (kg)	12.8 ± 1.4	12.9 ± 1.4	12.5 ± 0.9	12.9 ± 1.8	0.52
Cognitive development, 24 months (score)	9.22 ± 2.0	9.59 ± 1.2	9.56 ± 1.7	8.40 ± 2.7	0.16

Values are mean ± SD or %.

^a^Normal weight, overweight, and obese groups based on maternal pre-pregnancy BMI.

^b^Tests of significance between groups were calculated with analysis of variance.

^c^Tests of significance between groups were based on chi-square test.

**Fig 1:**
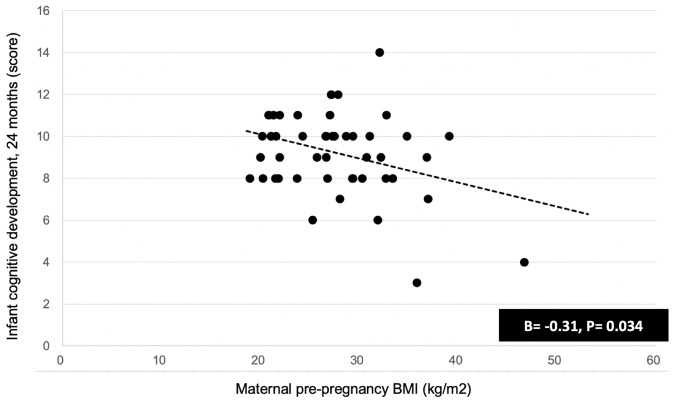
Maternal pre-pregnancy BMI related to infant cognitive development scores at 24 months. Association between maternal pre-pregnancy BMI with infant cognitive development scores at 24 months was examined using linear regression models, adjusting for maternal age, secretor status, education level, infant age, infant sex, and infant birth weight (scatter plots are unadjusted).

Maternal pre-pregnancy BMI predicted lower infant cognitive development score (β = -0.31, P = 0.03) (Fig 1). Maternal pre-pregnancy BMI was not associated with breast milk feeding frequency at 1 month (β = -0.08) and 6 months (β = -0.09) (both P> 0.05). Maternal pre-pregnancy BMI was not associated with any of the nineteen HMOs, including 2’FL at 1 month (β = -0.10) and 6 months (β = 0.01) (both P> 0.05). This indicated that maternal pre-pregnancy BMI was an independent predictor of infant cognitive development score.

**Fig 2:**
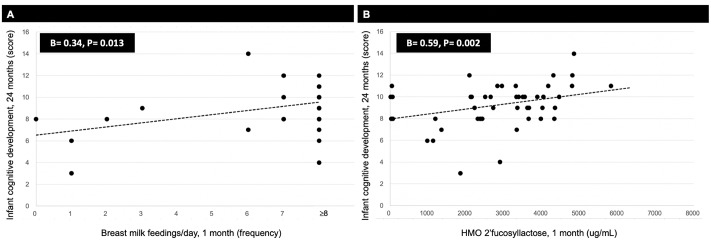
Breast milk feeding frequency and HMO 2’FL at 1 month related to infant cognitive development scores at 24 months. Associations between breast milk feeding frequency (A) and HMO 2’FL (B) at 1 month with infant cognitive development at 24 months were examined using linear regression models, adjusting for maternal age, pre-pregnancy BMI, secretor status, education level, infant age, infant sex, and infant birth weight (scatter plots are unadjusted).

**Fig 3:**
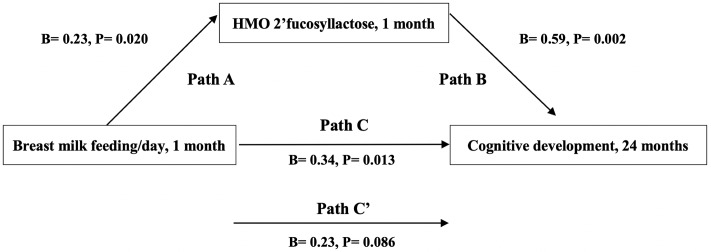
Mediation models of the pathway from breast milk feeding frequency at 1 month to infant cognitive development scores at 24 months via HMO 2’FL at 1 month. Path A is the path from breast milk feeding frequency (exposure) to 2’FL (mediator). Path B is the path from 2’FL (mediator) to infant cognitive development scores (outcome). Path C is the path from breast milk feeding frequency (exposure) to infant cognitive development scores (outcome). Path C’ is the path from breast milk feeding frequency (exposure) to infant cognitive development scores (outcome) when controlled for 2’FL (mediator). Standardized (B) coefficients with p values are presented. All models were adjusted for the following control variables: maternal age, pre-pregnancy BMI, secretor status, education level, infant age, infant sex, and infant birth weight.

Breast milk feeding frequency (β = 0.34) and HMO 2’FL (β = 0.59) at 1 month predicted higher infant cognitive development scores (both P≤ 0.01) (Fig 2). Breast milk feeding frequency also predicted greater 2’FL at 1 month (β = 0.23, P = 0.02). The association between breast milk feeding frequency at 1 month and higher infant cognitive development scores was no longer significant after further adjustment for 2’FL (Fig 3). Mediation analysis revealed that 2’FL at 1 month explained the association between breast milk feeding frequency at 1 month and infant cognitive development scores (estimation of mediation effect = 0.13, P = 0.04). This indicated that the association of breast milk feeding frequency at 1 month with higher infant cognitive development scores was explained by 2’FL. Findings were similar when the analysis was conducted in secretors only. In addition, linear regression models revealed that HMO DSLNT at 1 month predicted lower infant cognitive development scores (β = -0.32, P = 0.02). No other individual HMOs at 1 month were related to infant cognitive development scores.

Breast milk feeding frequency (β = 0.21) and HMO 2’FL (β = 0.30) at 6 months were not associated with infant cognitive development scores (both P> 0.05). However, linear regression models revealed that HMOs LNH (β = 0.57) and FLNH (β = 0.41) at 6 months predicted higher infant cognitive development scores (both P≤ 0.02). HMO LSTb at 6 months predicted lower infant cognitive development scores (β = -0.55, P< 0.01). No other individual HMOs at 6 months were related to infant cognitive development scores.

## Discussion

In this study of Hispanic participants, we found that maternal and breast milk characteristics were associated with infant cognitive development at 24 months of age, an outcome that prior studies have suggested tracks across the life course to mitigate risk for psychiatric disorders and maximize scholastic potential and vocation [3, 4, 6]. While maternal obesity was a separate and negative influence, the combination of breast milk feedings and HMOs was a positive influence to augment infant cognitive development. That is, greater frequency of breast milk feedings at 1 month contributed to infant cognitive development through greater exposure to HMO 2’FL at 1 month. Because similar associations were not observed at 6 months suggests that early exposure to 2’FL may be most important for enhancing infant learning and memory, with benefits that may endure into adulthood.

Mothers are the earliest influences of infant cognitive development, as mothers inform infant biology as well as infant environment and behavior. One maternal factor that impacts infant cognitive development is maternal weight status. In other studies, mothers who were overweight and obese before pregnancy (BMI ≥25 kg/m^2^) were more likely to have a child with lower intelligence test scores at 4 to 14 years of age [28–30]. Mothers with excess weight gain during pregnancy (>1 standard deviation) were more likely to have a child with lower verbal and non-verbal test scores at 10 years of age [30, 31]. Our finding that maternal obesity before pregnancy predicted lower infant cognitive development scores at 24 months has been reported previously [32, 33], but builds on existing evidence through our exclusive study of Hispanic participants. It lends even more support for clinical trials to target maternal obesity for the benefit of the infant, particularly in disparate cohorts.

As we postulated in a previous publication in the same cohort [19], maternal obesity may persist after pregnancy, and alter breast milk characteristics [8, 9, 19, 34]: this includes neuroactive components for infant cognitive development [9, 34]. However, we found that maternal obesity before pregnancy was a separate influence, unrelated to breast milk factors. This may be due to several reasons. First, maternal pre-pregnancy BMI is a marked influence of fetal brain development that supports infant cognitive development [35–37]. However, we can only speculate that intrauterine exposure to maternal weight status contributed to infant cognitive development in our cohort. Second, maternal pre-pregnancy BMI may not alter HMOs, as carbohydrates are relatively stable in human milk [38]. That said, there is some evidence that carbohydrates vary with breast milk feeding frequency [38].

Indeed, breast milk feeding frequency and HMOs were distinct determinants of infant cognitive development, with cumulative potential. Our data revealed that greater frequency of breast milk feedings at 1 month contributed to improved infant cognitive development. This in line with multiple studies to date: infants who ever breastfed tend to have better cognitive performance in childhood vs. those who never breastfed [5, 39]. However, we found that more frequent breast milk feedings at 6 months were not associated with infant cognitive development. Though similar results have been reported [39], there are discrepancies across studies in terms of optimal feeding time and duration [5]. This may be due to differences in study populations, assessment tools, and infant outcomes. It may also be due to a narrow research focus on breast milk amount vs. composition, as well as the timing of breast milk exposure. Because individual HMOs tend to be stable or decrease (vs. increase) over lactation, there may be a critical temporal window for maximizing infant cognitive development [38].

To this end, we found that the link between breast milk feeding frequency at 1 month and infant cognitive development was explained by HMO 2’FL. Though hundreds of HMOs have been identified, 2’FL has emerged in the context of one that relates to cognitive development. In animals, exposure to 2’FL enhanced cognitive performance in rat pups as well as adults [13]: this was through long-term potentiation of the brain hippocampus that facilitates spatial learning and memory [14]. Indeed, HMOs are a source of prebiotics to nourish the gut microbiome, and 2’FL may shape infant cognitive development through several mechanisms: 1) HMOs may diminish gut dysbiosis (imbalance) and consequent inflammation from the immature intestine to the developing brain, to protect against deficits in early cognition [15, 16]; and 2) HMOs may enhance production of metabolites that support infant cognitive development. In vitro, 2’FL increased abundance of gut microbes *Bacteroides* and *Lactobacillus*, and this increased production of short chain fatty acids, a substrate in brain signaling [17, 40]. It follows that a study in infants found that greater abundance of the same gut microbes was related to better cognitive performance at 2 years of age [18].

It is possible that individual HMOs DSLNT, LSTb, LNH, and FLNH were also associated with infant cognitive development through proliferation or depletion of specific gut microbes [41]. Indeed, DSLNT and LSTb are similar in structure [41], and may share a similar function that impacts poorer infant cognitive development, with differential effects at 1 and 6 months. However, compared to 2’FL, there is quite limited data on these prebiotic substrates to elucidate potential effects at this time. Future reports from this cohort will assess the influence of 2’FL and individual HMOs on infant cognitive development in combination with influences of the gut microbiome.

This study has several limitations. For example, the use of mediation models in a cross-sectional design would be strengthened with the addition of a randomized design, to eliminate possible confounders and better establish causality. Moreover, our results are specific to a small sample of Hispanic mother-infant pairs located in the Southwestern United States. The limited number of participants could have reduced statistical power to detect significant associations among exposure, outcome, and mediator variables, especially when data were dichotomized relative to maternal pre-pregnancy BMI categories (as in Table 1) [42, 43]. In addition, our cohort could be considered somewhat homogenous in socioeconomic status, education level, and cultural practices that surround food choice and eating behaviors, and this limits generalizability of our results. That said, Hispanic mother-infant pairs are at increased risk for obesity, and our findings offer more insight into racial/ethnic disparities [44].

In conclusion, results from this study demonstrate that maternal factors influence infant cognitive development through multiple mechanisms. Though maternal obesity may be a separate negative influence, greater breast milk feeding frequency at 1 month contributed to infant cognitive development, and this was explained through greater exposure to 2’FL. Because the association was not observed at 6 months indicates that early exposure to 2’FL may be a critical temporal window for positively influencing infant cognitive development. This information may guide interventions to improve maternal factors and feeding practices to optimize infant cognitive abilities and learning potential.

## Supporting information

S1 Fig Participant flow chart. (PDF)

S2 Fig HMO DSLNT at 1 month related to infant cognitive development scores at 24 months. Association between HMO DSLNT at 1 month with infant cognitive development at 24 months was examined using linear regression models, adjusting for maternal age, pre-pregnancy BMI, secretor status, education level, infant age, infant sex, and infant birth weight (scatter plots are unadjusted).
(TIFF)

S1 STROBE Checklist Strengthening the reporting of observational studies in epidemiology. (PDF)
