# Supplementary figures and images for: Human milk oligosaccharide 2’-fucosyllactose links feedings at 1 month to cognitive development at 24 months in infants of normal and overweight mothers
Source: PLoS One. 2020 Feb 12;15(2):e0228323. doi: 10.1371/journal.pone.0228323 (PMC7015316; doi:10.1371/journal.pone.0228323)

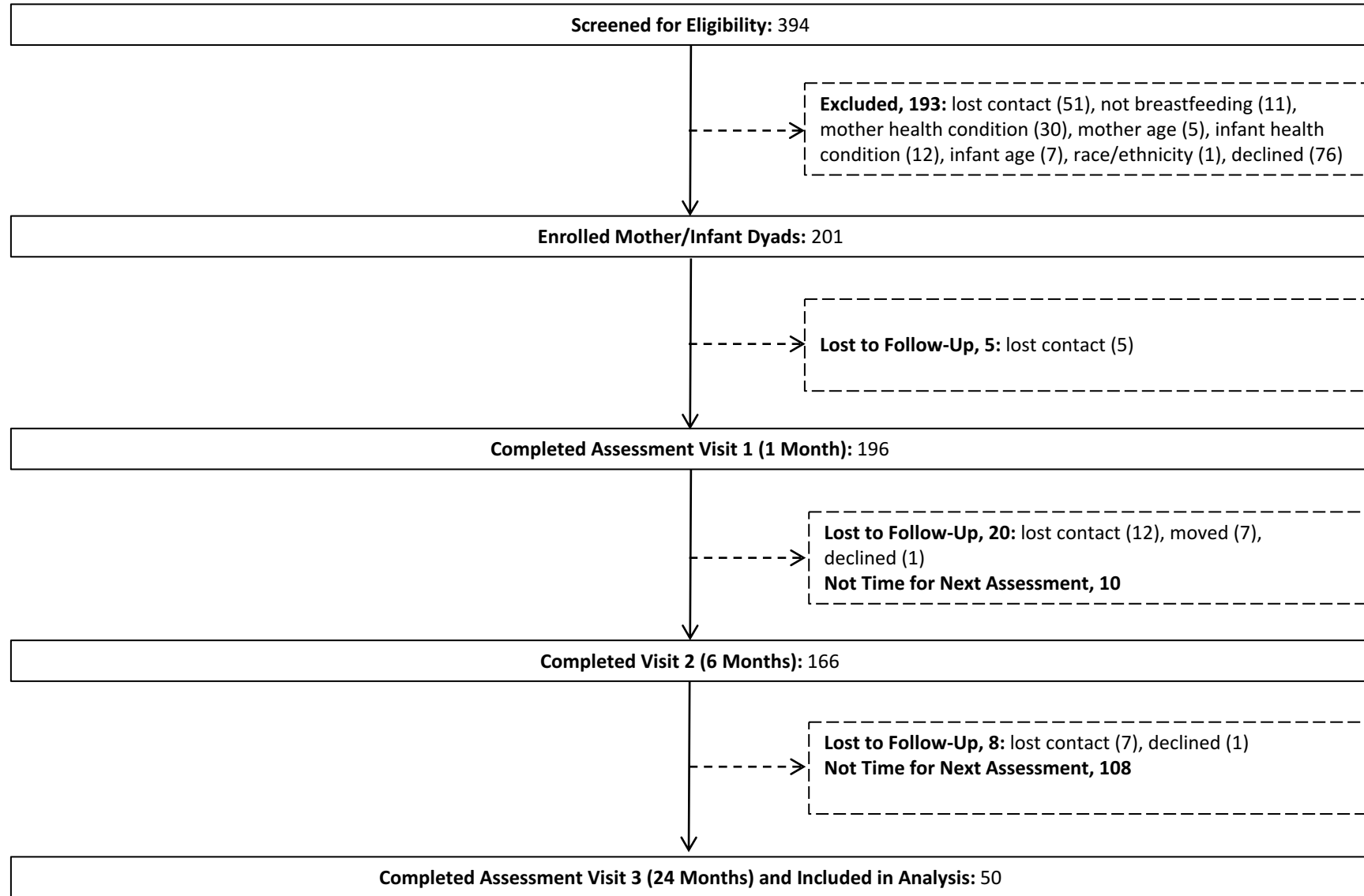

Supplement: S1 Fig — (PDF) [file pone.0228323.s001.pdf]

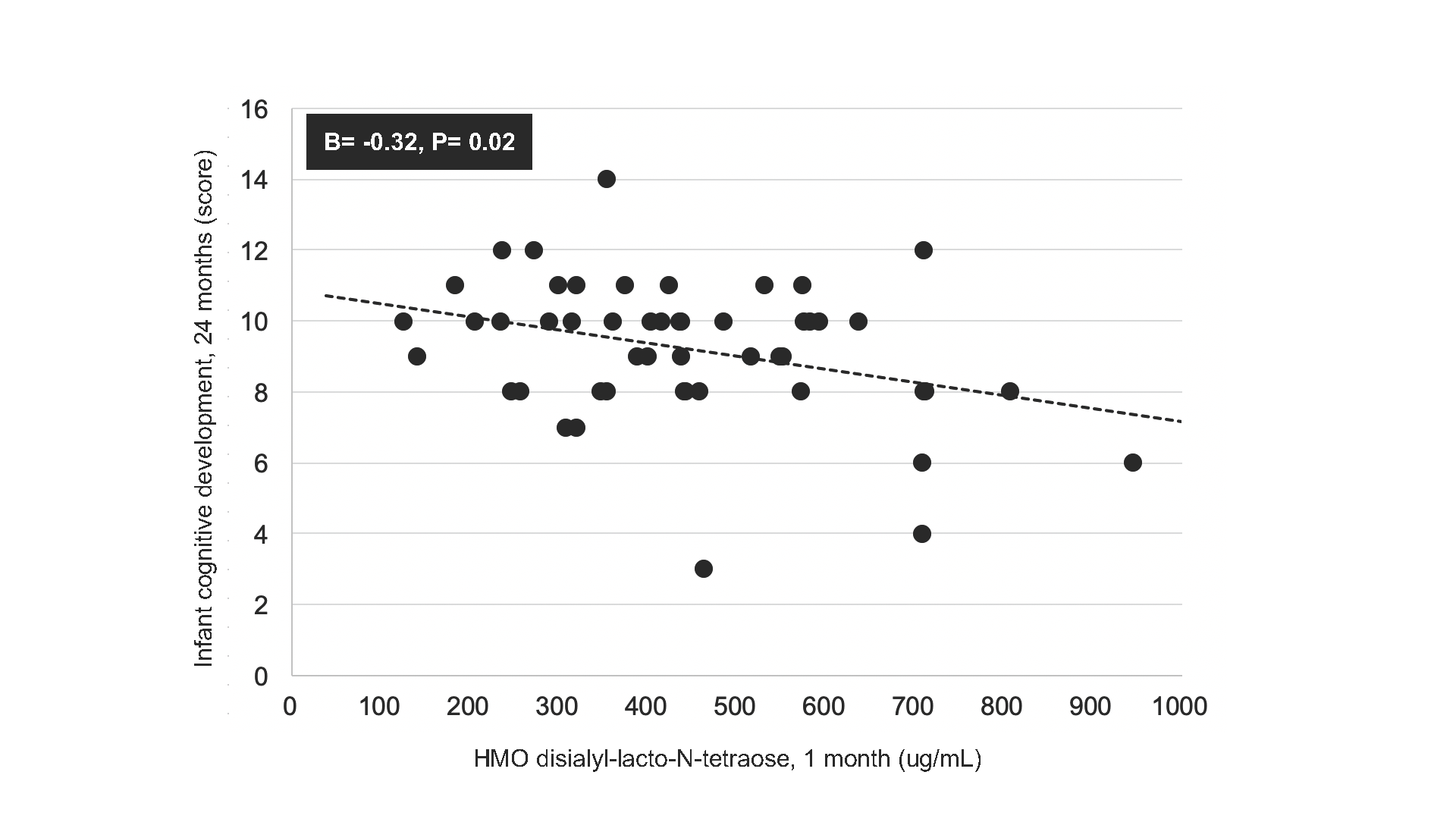

Supplement: S2 Fig — Association between HMO DSLNT at 1 month with infant cognitive development at 24 months was examined using linear regression models, adjusting for maternal age, pre-pregnancy BMI, secretor status, education level, infant age, infant sex, and infant birth weight (scatter plots are unadjusted). (TIFF) [file pone.0228323.s002.tiff]
